# Integrative Bioinformatic Analysis of Cellular Senescence Genes in Ovarian Cancer: Molecular Subtyping, Prognostic Risk Stratification, and Chemoresistance Prediction

**DOI:** 10.3390/biomedicines13040877

**Published:** 2025-04-04

**Authors:** Ailian Li, Dianbo Xu

**Affiliations:** Department of Gynecology, The Affiliated Jiangning Hospital of Nanjing Medical University, Nanjing 211199, China

**Keywords:** ovarian cancer, cellular senescence, bioinformatic analysis, prognosis, tumor microenvironment, consistency clustering

## Abstract

**Background**: Ovarian cancer (OC) is a heterogeneous malignancy associated with a poor prognosis, necessitating robust biomarkers for risk stratification and therapy optimization. Cellular senescence-related genes (CSGs) are emerging as pivotal regulators of tumorigenesis and immune modulation, yet their prognostic and therapeutic implications in OC remain underexplored. **Methods**: We integrated RNA-sequencing data from TCGA-OV (n = 376), GTEx (n = 88), and GSE26712 (n = 185) to identify differentially expressed CSGs (DE-CSGs). Consensus clustering, Cox regression, LASSO-penalized modeling, and immune infiltration analyses were employed to define molecular subtypes, construct a prognostic risk score, and characterize tumor microenvironment (TME) dynamics. Drug sensitivity was evaluated using the Genomics of Drug Sensitivity in Cancer (GDSC)-derived chemotherapeutic response profiles. **Results**: Among 265 DE-CSGs, 31 were prognostic in OC, with frequent copy number variations (CNVs) in genes such as STAT1, FOXO1, and CCND1. Consensus clustering revealed two subtypes (C1/C2): C2 exhibited immune-rich TME, elevated checkpoint expression (PD-L1, CTLA4), and poorer survival. A 19-gene risk model stratified patients into high-/low-risk groups, validated in GSE26712 (AUC: 0.586–0.713). High-risk patients showed lower tumor mutation burden (TMB), immune dysfunction, and resistance to Docetaxel/Olaparib. Six hub genes (HMGB3, MITF, CKAP2, ME1, CTSD, STAT1) were independently predictive of survival. **Conclusions**: This study establishes CSGs as critical determinants of OC prognosis and immune evasion. The molecular subtypes and risk model provide actionable insights for personalized therapy, while identified therapeutic vulnerabilities highlight opportunities to overcome chemoresistance through senescence-targeted strategies.

## 1. Introduction

Ovarian cancer (OC) ranks as the fifth leading cause of cancer-related mortality among women worldwide, with over 313,000 new cases annually and a five-year survival rate stagnating below 46% [1,2]. Late-stage diagnoses and intrinsic molecular heterogeneity contribute to therapeutic resistance and poor prognosis, underscoring the urgent need for biomarkers guiding personalized therapy [3]. While genomic studies have implicated dysregulated proliferation and apoptosis in OC pathogenesis [4], the role of cellular senescence—a state of irreversible cell cycle arrest—remains poorly defined, despite its dualistic role in both suppressing tumorigenesis and fostering pro-metastatic microenvironments [5,6,7,8,9].

Cellular senescence is triggered by diverse stressors, including DNA damage, oxidative stress, and telomere attrition [5,6]. Senescent cells secrete pro-inflammatory factors via the senescence-associated secretory phenotype (SASP), remodeling the tumor microenvironment (TME) to promote immune evasion, angiogenesis, and chemoresistance [7,8,9]. In OC, preclinical models suggest that senescent tumor-associated fibroblasts (TAFs) drive immunosuppression through SASP-mediated recruitment of myeloid-derived suppressor cells (MDSCs) [10,11,12]. Paradoxically, senescence induction in cancer cells may also enhance chemosensitivity, highlighting context-dependent roles that warrant systematic exploration [13].

Despite these advances, no study has comprehensively mapped the landscape of senescence-related genes (CSGs) in OC or evaluated their clinical utility for prognosis prediction and therapy selection. This study aims to (1) identify CSGs with prognostic significance through multi-omics data integration; (2) delineate CSG-driven molecular subtypes and their immune microenvironment features; and (3) uncover CSG-associated therapeutic vulnerabilities using drug sensitivity profiling. Our findings provide a framework for targeting senescence-immune crosstalk to overcome treatment resistance in OC.

## 2. Materials and Methods

### 2.1. Data Acquisition and Preprocessing

**Figure 1:**
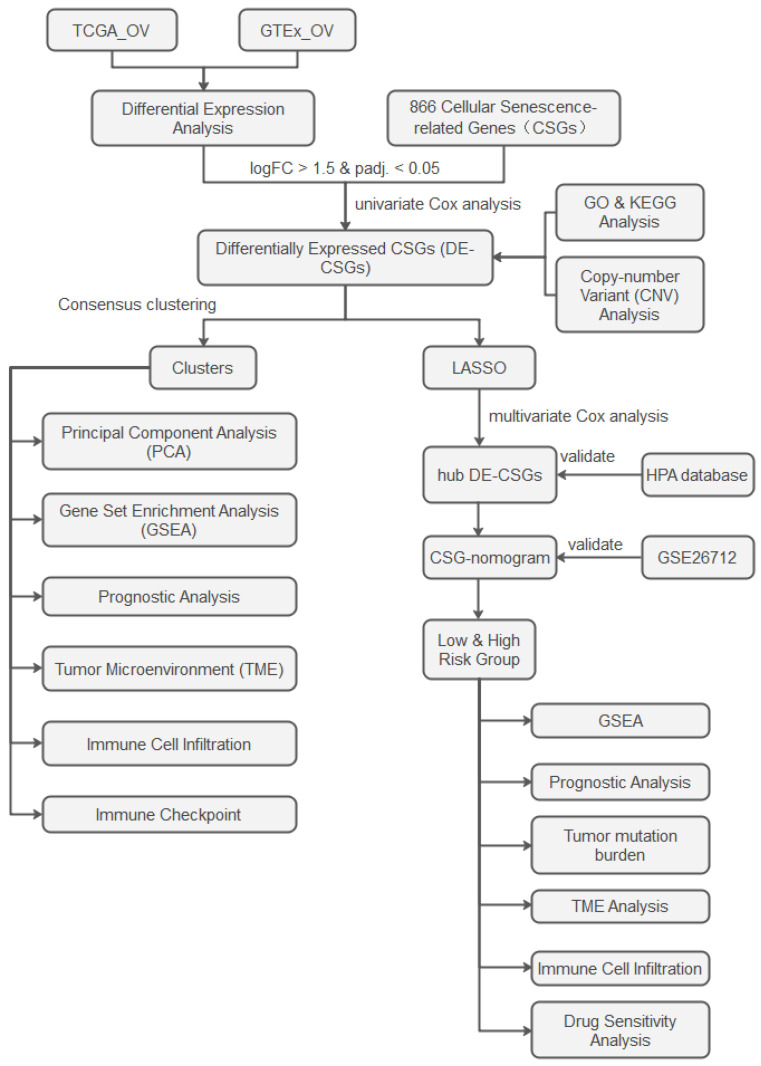
Study flowchart.

The analytical workflow is illustrated in Figure 1. RNA-sequencing raw count matrices from 379 OC samples (TCGA-OV) and 88 normal ovarian tissues (GTEx) were downloaded from the UCSC Xena platform [14]. After merging the datasets and removing duplicate genes, we unified gene symbols to Ensembl IDs, retaining only intersecting genes across both platforms. Samples with incomplete clinical annotations were excluded, retaining 376 OC cases for downstream analyses. To address batch effects inherent in integrating distinct genomic datasets (TCGA vs. GTEx), raw counts were harmonized using the ComBat-seq algorithm [15]. Normalization was then performed via DESeq2 [16]: (1) median-of-ratios normalization for library size adjustment. (2) Variance-stabilizing transformation (VST) to stabilize expression variance across dynamic ranges. Somatic mutation spectra, copy number variation (CNV) landscapes, and clinicopathological metadata were concurrently extracted from TCGA. An independent validation cohort (GSE26712, n = 185 serous ovarian carcinoma patients) was procured from the GEO database. We obtained 866 CSGs from the CellAge database (Table S1), which exclusively includes genes with experimental evidence of regulating cellular senescence [17]. This ensures high-confidence selection of biologically relevant targets.

### 2.2. Differential Expression Profiling

To delineate senescence-associated transcriptional alterations, we applied the limma framework [18] for comparative analysis between OC and normal tissues, adopting thresholds of |log2FoldChange (FC)| > 1.5 and adjusted *p* < 0.05. Intersection of differentially expressed genes (DEGs) with the CSGs identified senescence-related DEGs (DE-CSGs). Prognostically significant DE-CSGs were further selected through univariate Cox regression (*p* < 0.05). Functional annotation of these genes leveraged Gene Ontology (GO) profiling and Kyoto Encyclopedia of Genes and Genomes (KEGG) enrichment analyses (“ClusterProfiler” (version 4.10.1) and “org.Hs.eg.db” (version 3.18.0) packages) [19], complemented by genome-wide CNV aberration mapping.

### 2.3. Molecular Subtyping Driven by Senescence Signatures

Consensus clustering (“ConsensusClusterPlus” package (version 1.66.0), K-means algorithm) partitioned OC patients into senescence-defined subtypes [20]. Inter-subtype transcriptomic divergence was validated via principal component analysis (PCA). Subsequent Gene Set Enrichment Analysis (GSEA) (“ClusterProfiler” package) using the C2 curated gene sets (MSigDB c2.cp.all.v2022.1.Hs) decoded pathway-level disparities, while survival disparities were quantified using Kaplan–Meier (K–M) curves (“survival” (version 3.5.8), and “survminer” (version 0.4.9) packages). The ESTIMATE algorithm deconvoluted TME scores [21], and ssGSEA (“GSVA” package (version 1.50.5)) enumerated infiltrating immune cell fractions across subtypes [22]. Immune checkpoint genes expression patterns were comparatively assessed.

### 2.4. Prognostic Model Construction

The least absolute shrinkage and selection operator (LASSO)-penalized Cox regression (“glmnet” package (version 4.1.8)) with 10-fold cross-validation was applied to select optimal DE-CSGs and construct a composite risk score: risk score = ∑ (expr_genei × coefficient_genei). Patients were dichotomized into high-/low-risk cohorts by median score. Survival disparities were evaluated via log-rank testing (“survminer” package), with risk–clinicopathological correlations visualized through “ggplot2”.

### 2.5. Hub Genes Identification and Nomogram Construction

A multivariable Cox regression analysis identified six hub CSGs (HMGB3, MITF, CKAP2, ME1, CTSD, STAT1) as independent prognostic factors for overall survival (OS) (*p* < 0.05). These were integrated into a nomogram (“rms” package (version 6.7.1)) predicting 1-, 3-, and 5-year survival probabilities. The model discrimination was evaluated through time-dependent operating characteristic (ROC) and calibration curves, with external validation performed in GSE26712.

### 2.6. Functional and Genomic Characterization of Risk Groups

GSEA was performed to identify significantly enriched biological pathways distinguishing high- and low-risk groups. Tumor mutation burden (TMB) was quantified via “maftools” package (version 2.18.0), correlating mutational landscapes with risk stratification.

### 2.7. Immune Infiltration

The ssGSEA algorithm was employed to quantify enrichment scores of 24 functionally distinct immune cell populations across risk-stratified cohorts, while ESTIMATE analysis was concurrently performed to compute comprehensive stromal and immune scores characterizing the TME landscape in ovarian cancer patients.

### 2.8. Drug Sensitivity Analysis

Pharmacogenomic analysis utilizing the Genomics of Drug Sensitivity in Cancer (GDSC) database (https://www.cancerrxgene.org/, accessed on 27 December 2024) was conducted with “oncoPredict” (version 0.2) and “pRRophetic” (version 0.5) R packages to quantify therapeutic responses through half-maximal inhibitory concentration (IC50) measurements [23,24]. Comparative analysis between risk-stratified cohorts revealed ovarian cancer agents with distinct drug sensitivity profiles.

### 2.9. Statistical Methods

The statistical analysis was implemented in R (version 4.3.3). Continuous variables with normal distribution were analyzed using Student’s *t*-test, while non-parametric comparisons employed the Wilcoxon rank-sum test. Categorical variable analyses utilized χ² tests or Fisher’s exact tests as appropriate. Data visualization was created with “ggplot2” (version 3.5.1), “ggpubr” (version 0.6.0), and “enrichplot” (version 1.20.0) packages.

## 3. Results

### 3.1. Transcriptomic Landscape of DE-CSGs

**Figure 2:**
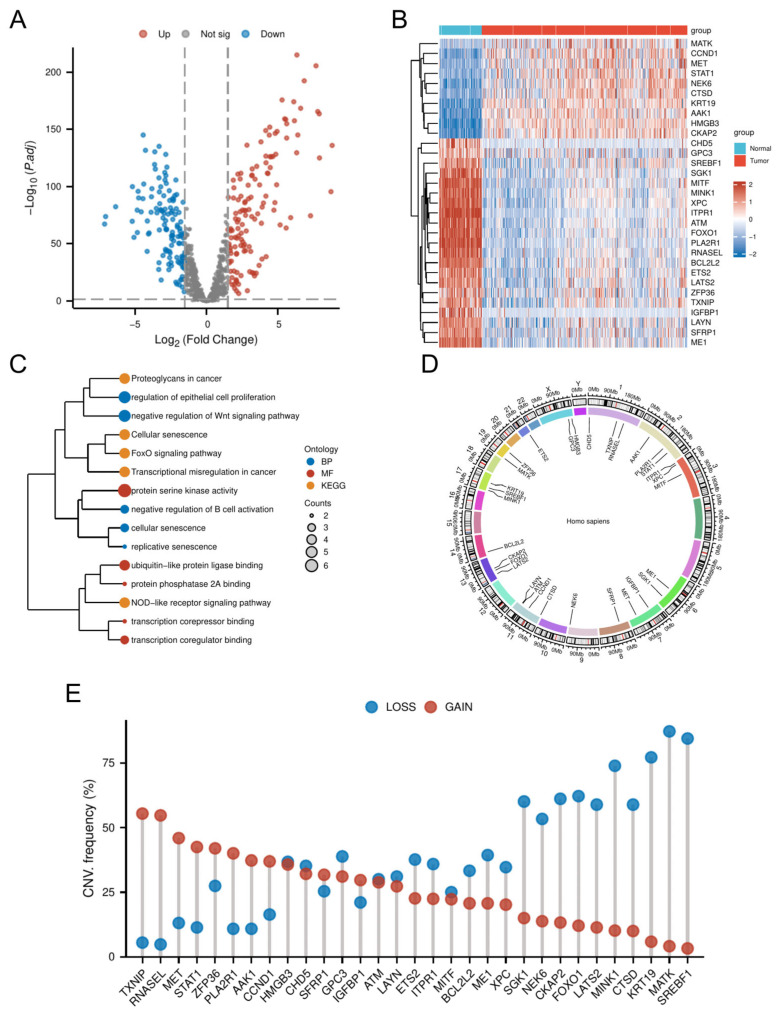
Genomic and transcriptomic profiling of DE-CSGs. (**A**) Volcano plot of DE-CSGs between tumor and normal tissues. (**B**) Heatmap showing expression patterns of 31 prognostic DE-CSGs. (**C**) Functional enrichment analysis of DE-CSGs using GO and KEGG. BP: biological process; MF: molecular function. (**D**) Chromosomal distribution of DE-CSGs. (**E**) CNV frequency analysis of DE-CSGs in OC.

Comparative transcriptomic analysis of OC tissues versus normal tissues identified 4247 DEGs. Intersection of these DEGs with the 866 CSGs yielded 265 DE-CSGs, including 134 upregulated and 131 downregulated candidates (Figure 2A and Table S2). Univariate Cox regression analysis identified 31 DE-CSGs significantly associated with OS in the TCGA cohort, whose distinct expression profiles between OC and normal ovarian tissues are visually demonstrated in the hierarchically clustered heatmap (Figure 2B). Functional annotation of the 31 prognostic DE-CSGs revealed distinct pathway preferences (Figure 2C). Biological processes centered on immunoregulation (B cell activation suppression), developmental signaling modulation (Wnt pathway inhibition), and senescence dynamics (epithelial proliferation control, replicative/cellular senescence). Molecular functions predominantly involved transcriptional regulation (coregulator/corepressor binding) and post-translational modification machinery (serine kinase/PP2A phosphatase activity, ubiquitin-like ligase interactions). KEGG pathway enrichment underscored their roles in cancer biology through three axes: signal transduction (FoxO and NOD-like receptor pathways), senescence and microenvironment modulation (cellular senescence, proteoglycan networks), and oncogenic transformation (transcriptional deregulation).

### 3.2. CNV Analysis of DE-CSGs

Genomic instability analysis of the 31 prognostic DE-CSGs revealed clinically relevant copy number alterations (Figure 2D,E). CNV data profiling identified recurrent genomic events, with chromosome 1q (TXNIP, RNASEL), 2q (STAT1, PLA2R1), 7q (MET), and 19q (ZFP36) harboring recurrent amplifications (frequency > 40%), while 13q (FOXO1, CKAP2), 17q (MINK1, KRT19, SREBF1), and 19q (MATK) exhibited prevalent deletions (frequency > 50%). These loci represent potential therapeutic vulnerabilities linked to OC pathogenesis.

### 3.3. Cellular Senescence-Driven Molecular Subtyping

**Figure 3:**
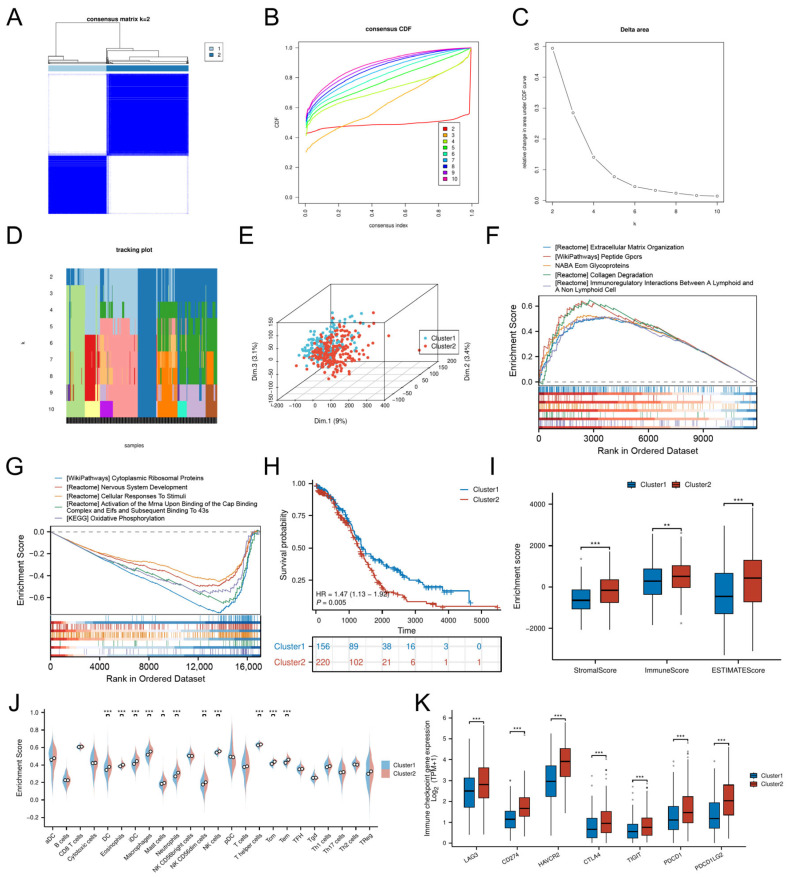
Molecular subtyping based on DE-CSGs. (**A**) Consensus clustering analysis defining two molecular subtypes (k = 2). (**B**) Cumulative distribution function (**C**,**D**,**F**) curves for cluster number determination. (**C**) Delta area analysis for optimal cluster selection. (**D**) Tracking plot validation of cluster stability. (**E**) PCA visualization of subtype separation. (**F**,**G**) GSEA of biological pathways in (**F**) C1 and (**G**) C2 subtypes. (**H**) K–M survival curves comparing OS between subtypes. (**I**,**J**) TME scores and immune cell infiltration levels across subtypes. (**K**) Differential expression of immune checkpoint molecules between subtypes. (* *p* < 0.05; ** *p* < 0.01; *** *p* < 0.001).

Consensus clustering of DE-CSGs stratified OC patients into two molecular subtypes, Cluster 1 (C1, n = 156) and Cluster 2 (C2, n = 220) (Table S3), with optimal cluster stability at k = 2 (Figure 3A–D). PCA confirmed distinct transcriptional landscapes between subtypes (Figure 3E). The GSEA revealed subtype-specific pathway activation: the C2 subtype exhibited immune–stromal crosstalk involving extracellular matrix (ECM) glycoproteins, collagen degradation, and lymphoid-non-lymphoid cell interactions, while C1 was enriched for cytoplasmic ribosomal proteins and oxidative phosphorylation (Figure 3F,G).

Despite heightened infiltration of dendritic cells (DCs, iDCs), eosinophils, macrophages, mast cells, neutrophils, NK cells (CD56dim, CD56bright), and T lymphocytes (T helper, central memory T cells [Tcm], effector memory T cells [Tem]) in C2 (Figure 3J), this subtype demonstrated significantly worse OS (HR = 1.47, *p* = 0.005; Figure 3H) and lower stromal/immune/ESTIMATE scores compared to C1 (Figure 3I). The adverse prognosis of C2 correlated with coordinated upregulation of immune checkpoint molecules, including LAG3, HAVCR2 (TIM-3), CD274 (PD-L1), CTLA4, PDCD1LG2 (PD-L2), TIGIT, and PDCD1 (PD-1) (Figure 3K), suggesting potential immune evasion mechanisms.

To evaluate the relationship between CSG subtypes and TCGA classifications [25], we generated a Sankey diagram illustrating the distribution of C1/C2 tumors across TCGA subtypes (Figure S1). C2 tumors were evenly distributed among all four TCGA categories (Proliferative: 23.5%, Immunoreactive: 22.4%, Differentiated: 26.4%, Mesenchymal: 27.7%), yet exhibited uniformly poor survival in the overall cohort. Stratified survival analysis within individual TCGA subtypes revealed no significant differences between C1 and C2 (all *p* > 0.15, Figure S2), suggesting that the prognostic power of CSG subtyping arises from its ability to transcend—rather than refine—traditional molecular categories.

### 3.4. Identification and Validation of Hub CSGs

**Figure 4:**
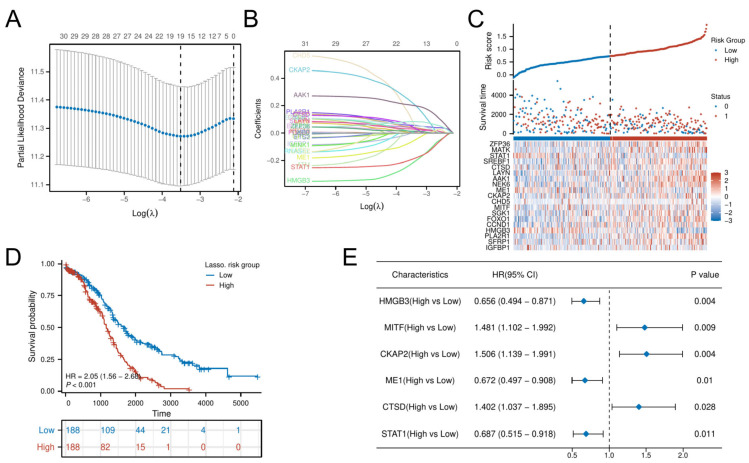
Construction of the risk score model and identification of the hub CSGs. (**A**) LASSO coefficient profiles for feature selection. (**B**) Partial likelihood deviance curve for optimal λ selection. (**C**) Distribution of risk scores across patients. (**D**) K–M analysis of survival outcomes by risk group. (**E**) Multivariate Cox regression analysis of prognostic DE-CSGs.

**Figure 5:**
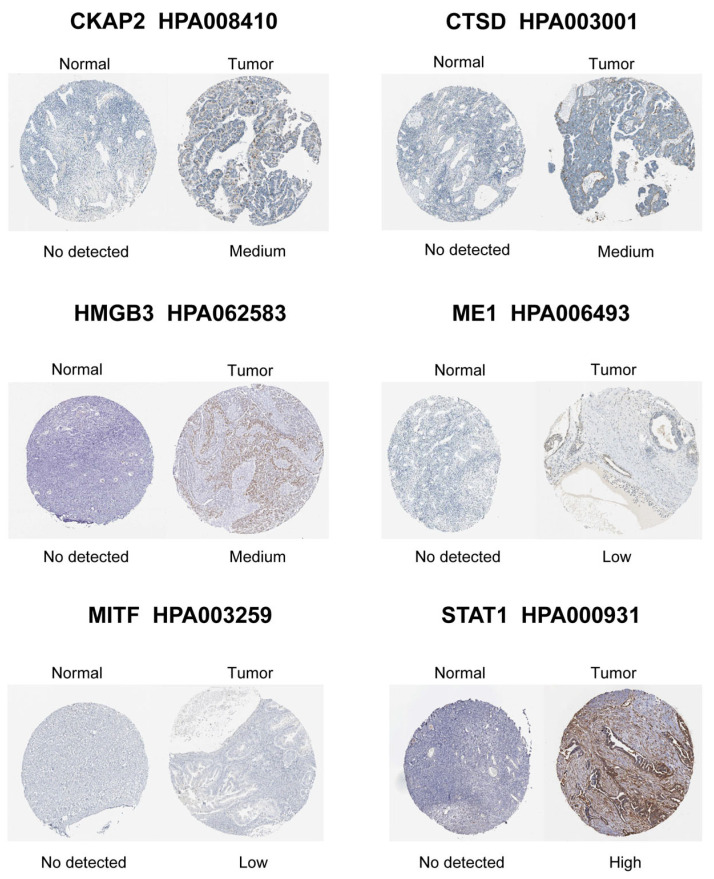
Immunohistochemical staining validation of hub gene protein expression using the HPA database.

LASSO-penalized Cox regression distilled 19 prognostic DE-CSGs from the initial 31 candidates: IGFBP1, SFRP1, PLA2R, HMGB3, CCND1, FOXO1, SGK1, MITF, CHD5, CKAP2, ME1, NEK6, AAK1, LAYN, CTSD, SREBF1, STAT1, MATK, ZFP36 (Figure 4A,B). Patients were dichotomized into high-risk (n = 188) and low-risk (n = 188) cohorts by median risk score (Figure 4C). The high-risk group exhibited significantly poorer OS compared to the low-risk group (HR = 2.05, *p* < 0.001; Figure 4D). Multivariable Cox analysis prioritized six hub genes—HMGB3, MITF, CKAP2, ME1, CTSD, STAT1—as independent OS predictors (all *p* < 0.05; Figure 4E). Immunohistochemical validation via the Human Protein Atlas (HPA) confirmed significant overexpression of these hub genes in OC tissues versus normal controls (Figure 5).

### 3.5. Nomogram Development and External Validation

**Figure 6:**
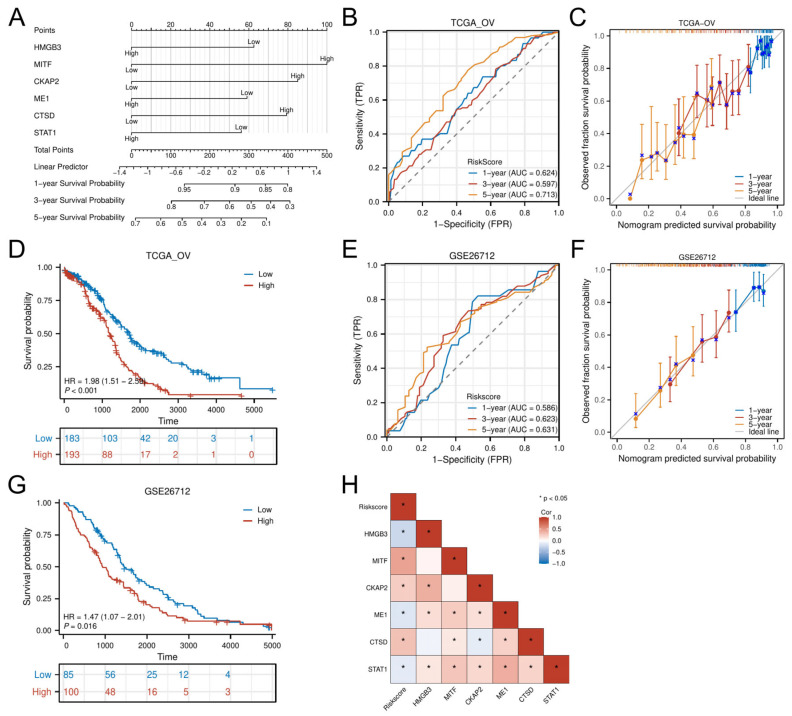
Development and evaluation of the prognostic nomogram. (**A**) Nomogram integrating risk scores for 1-, 3-, and 5-year survival prediction. (**B**,**C**) Time-dependent ROC curves and calibration plots in the training cohort. (**E**,**F**) External validation of the nomogram in the GSE26712 cohort. (**D**,**G**) K–M analysis by risk stratification in training and validation sets. (**H**) Correlation heatmap between hub genes and risk scores. * *p* < 0.05.

A multigene-integrated nomogram was constructed to predict OC outcomes, incorporating the six hub genes (Figure 6A). Time-dependent ROC analysis demonstrated moderate predictive accuracy in the TCGA-OV cohort, with AUC values of 0.624 (1 year), 0.597 (3 years), and 0.713 (5 years) (Figure 6B). Calibration curves confirmed close alignment between predicted and observed survival probabilities across all timepoints (Figure 6C). Multivariable Cox-derived risk stratification robustly discriminated patient outcomes, with the high-risk group showing significantly reduced OS (HR = 1.98, *p* < 0.001; Figure 6D).

External validation using the GSE26712 cohort replicated these findings, achieving comparable AUCs (1-year: 0.586; 3-year: 0.623; 5-year: 0.631) (Figure 6E) and well-calibrated prediction curves (Figure 6F). Survival patterns remained consistent, with high-risk patients demonstrating poorer prognosis (HR = 1.47, *p* = 0.016; Figure 6G). Correlation analysis further linked the six hub genes to risk scores: MITF, CKAP2, and CTSD showed positive associations (*p* < 0.05), while HMGB3, ME1, and STAT1 displayed negative correlations (*p* < 0.05) (Figure 6H).

### 3.6. Mutational Landscape and TMB-Driven Prognostic Stratification

**Figure 7:**
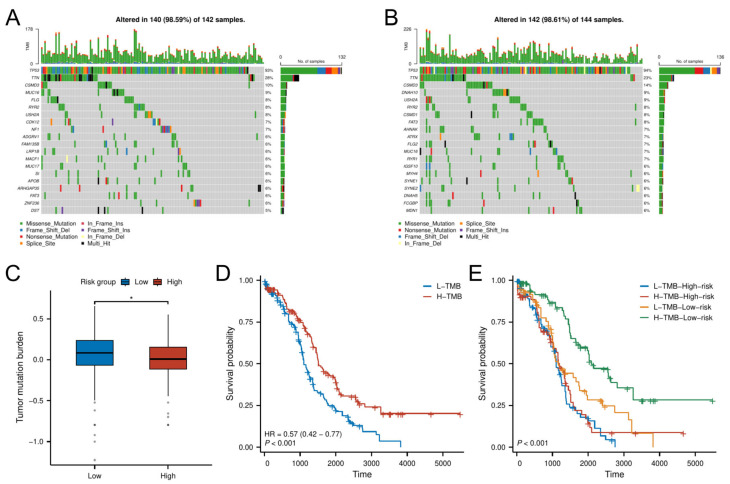
TMB analysis. (**A**,**B**) Mutational landscape of (**A**) high-risk and (**B**) low-risk groups. (**C**) TMB differences between risk groups. (**D**,**E**) K–M analysis stratified by TMB levels and combined risk–TMB status.

Analysis of somatic mutation profiles from the TCGA cohort revealed distinct mutational landscapes between risk groups (Figure 7A,B). The low-risk group exhibited a significantly higher TMB compared to the high-risk group (*p* < 0.05; Figure 7C).

Survival analysis demonstrated that patients with high TMB had better OS than those with low TMB (Figure 7D). Further stratification combining TMB and risk classification showed that the low-risk group with high TMB had the most favorable prognosis, outperforming all other subgroups (Figure 7E). This synergistic effect suggests TMB may enhance prognostic discrimination within the senescence-related risk framework.

### 3.7. Functional Pathway Enrichment in Risk Groups

**Figure 8:**
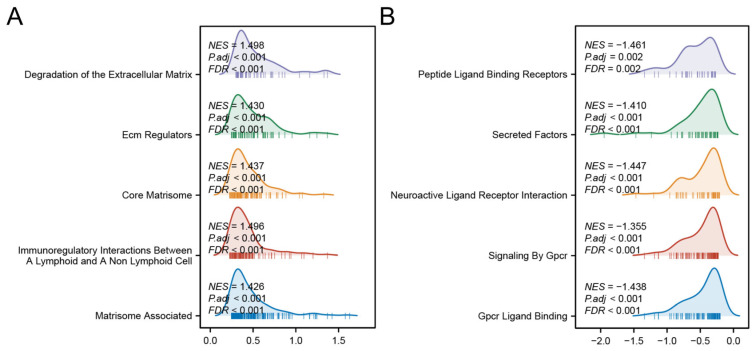
GSEA analyses of high- (**A**) and low-risk group (**B**).

GSEA revealed distinct biological pathways associated with each risk group. The high-risk group was significantly enriched in pathways related to ECM protein regulation, ECM degradation, and immune modulation between lymphoid and non-lymphoid cells (Figure 8A). In contrast, the low-risk group showed prominent activation of G-protein-coupled receptor (GPCR) ligand binding, GPCR signaling pathways, and interactions between neuroactive ligands and receptors (Figure 8B). These findings collectively suggest that the high-risk phenotype is driven by stromal co-option and immune evasion, whereas low-risk tumors may retain GPCR-mediated growth control and neuronal signaling-mediated tumor surveillance.

### 3.8. Immune Landscape and TME Characteristics

**Figure 9:**
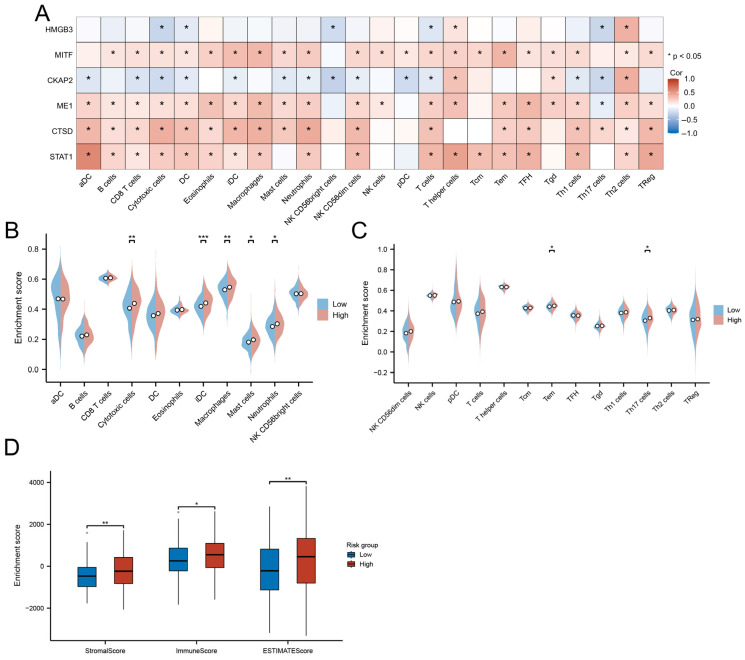
Immune microenvironment characterization. (**A**) Correlation between hub genes and immune cell infiltration. (**B**,**C**) Immune cell abundance differences between risk groups. (**D**) Comparative TME scores across risk groups. (* *p* < 0.05; ** *p* < 0.01; *** *p* < 0.001).

Correlation analysis of hub gene expression with immune infiltration patterns revealed distinct immunobiological associations. STAT1, CTSD, ME1, and MITF exhibited broad positive correlations with cytotoxic effectors (CD8+ T/NK cells), antigen-presenting cells (activated/immature DCs), and granulocytic lineages (neutrophils/mast cells). In contrast, CKAP2 showed significant negative associations with these immune populations (*p* < 0.05) but demonstrated selective positive correlation with Th2-polarized lymphocytes (Figure 9A).

The high-risk group displayed enhanced infiltration of functionally divergent immune subsets, including cytotoxic T cells (effector memory, Th17), myeloid cells (neutrophils, macrophages), and iDCs (*p* < 0.05; Figure 9B,C). Paradoxically, these immunologically active tumors concurrently exhibited elevated stromal remodeling activity, as evidenced by significantly higher ESTIMATE scores (immune: *p* < 0.05; stromal: *p* < 0.01) compared to low-risk counterparts (Figure 9D).

### 3.9. Risk-Stratified Drug Sensitivity Patterns

**Figure 10:**
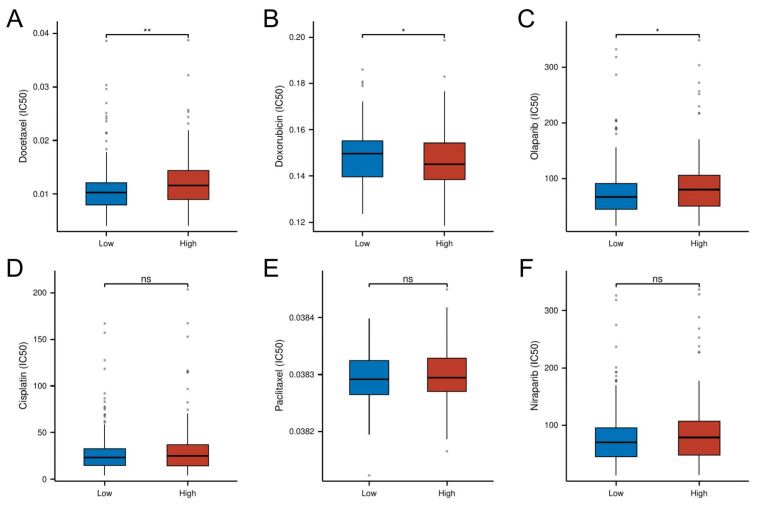
Differential drug response profiles between risk-stratified OC patients. (**A**) IC50 of Docetaxel. (**B**) IC50 of Doxorubicin. (**C**) IC50 of Olaparib. (**D**) IC50 of Cisplatin. (**E**) IC50 of Paclitaxel. (**F**) IC50 of Niraparib. (* *p* < 0.05; ** *p* < 0.01; ns, not significant)

Pharmacogenomic analysis revealed distinct drug sensitivity patterns between risk subgroups, with high-risk tumors demonstrating increased resistance to conventional chemotherapeutics. Specifically, the high-risk group exhibited significantly higher IC50 values for Docetaxel (*p* < 0.01) and the PARP inhibitor Olaparib (*p* < 0.05), suggesting potential cross-resistance to microtubule-targeting agents and DNA damage response inhibitors. Conversely, the low-risk group showed enhanced chemoresistance to doxorubicin (*p* < 0.05). No significant intergroup differences were observed for cisplatin, paclitaxel, or the second-generation PARP inhibitor niraparib (*p* > 0.05; Figure 10). These findings position the cellular senescence-related risk model as a predictive biomarker for optimizing chemotherapy selection.

## 4. Discussion

This study presents the first multi-omics integration to delineate the molecular features of CSGs in OC, uncovering their roles in TME heterogeneity, metabolic adaptation, and clinical outcomes. We identified CSG-driven molecular subtypes (C1/C2) with distinct survival patterns and constructed a multi-gene risk model highlighting therapeutic vulnerabilities.

Cellular senescence exhibits dualistic roles in cancer progression that are highly context-dependent. While acute senescence acts as a tumor-suppressive mechanism by irreversibly arresting damaged cells, chronic senescence promotes tumorigenesis through SASP mediated microenvironment remodeling [26]. Key senescence regulators maintain genomic stability in pre-malignant lesions [27], whereas SASP factors facilitate angiogenesis, immune evasion, and therapy resistance in established tumors [28]. In ovarian cancer, this duality is particularly complex due to unique peritoneal dissemination patterns and stromal crosstalk. Our work bridges this gap by linking CSGs to prognostic stratification and therapy resistance, positioning them as novel biomarkers for personalized treatment strategies.

The identification of 265 DE-CSGs, including 31 prognostic candidates, underscores the functional relevance of senescence pathways in OC biology. Notably, GO enrichment revealed strong associations with Wnt signaling inhibition—a pathway critical for stemness maintenance and differentiation [29]. This suggests that CSG-mediated Wnt suppression may enforce senescence barriers against malignant transformation, while its dysregulation could license senescence bypass, a hallmark of OC aggressiveness [30]. Molecular function analyses highlighted protein kinase activity modulation, aligning with evidence that kinase-driven signaling cascades govern senescence entry and escape [31]. KEGG pathway analysis highlighted enrichment in the FoxO signaling pathway, a key regulatory hub for cellular stress, senescence, and apoptosis [32]. The observed FoxO dysregulation may represent a molecular switch enabling OC cells to evade growth arrest, thereby offering a therapeutic leverage point.

CNV profiling of DE-CSGs uncovered genomic instability hotspots linked to OC pathogenesis. High-frequency amplifications (e.g., TXNIP, RNASEL) and deletions (e.g., MATK, SREBF1) suggest selective pressure to modulate senescence-associated proliferation control and metabolic adaptation [33,34]. For instance, TXNIP amplification may hyperactivate redox-sensitive senescence checkpoints, while SREBF1 loss could disrupt lipid homeostasis, creating a permissive niche for tumor survival [35,36].

By integrating Consensus Clustering with the K-means algorithm, we stratified the TCGA-OV cohort into two molecular subtypes (C1 and C2) with distinct clinical outcomes. Patients in the C2 subtype exhibited significantly shorter OS, suggesting a molecular phenotype conducive to aggressive tumor progression. The even distribution of C2 tumors across TCGA subtypes, combined with their consistent poor prognosis, indicates that CSG subtyping identifies a pan-TCGA high-risk phenotype driven by senescence-immune interactions. This phenotype may override subtype-specific biology, regardless of their TCGA classification. Clinically, this suggests that CSG subtyping could guide therapeutic strategies for high-risk patients overlooked by TCGA classification alone. GSEA revealed that C2 tumors were enriched in pathways associated with extracellular matrix (ECM) remodeling and immune regulation, indicating dual mechanisms of microenvironmental adaptation and immune evasion. For instance, ECM glycoprotein alterations may foster a tumor-supportive niche [37,38], while immune dysregulation—marked by upregulated checkpoint molecules such as PD-L1 and CTLA-4—could enable tumor cells to escape immune surveillance [39,40]. In contrast, the C1 subtype was linked to pathways involving cytosolic ribosomal proteins and oxidative phosphorylation, reflecting a metabolic state associated with restrained tumor aggressiveness [41]. TME analysis further corroborated these differences: C1 displayed lower immune, stromal, and ESTIMATE scores, indicative of an “immune-cold” microenvironment with limited immune infiltration and tumor-stroma interactions. Conversely, C2 exhibited pronounced infiltration of DCs, eosinophils, and cytotoxic lymphocytes, coupled with heightened expression of immune checkpoint genes, forming an immunosuppressive network. Although robust immune infiltration in C2 reflects host antitumor responses [42], concurrent checkpoint activation likely impairs effector cell functionality, permitting tumor persistence [39,40]. These findings underscore the therapeutic potential of immune checkpoint inhibitors for C2 patients, offering a strategy to alleviate immunosuppression and enhance antitumor immunity [43].

Our integrated analysis identified six hub CSGs (HMGB3, MITF, CKAP2, STAT1, ME1, CTSD) that exhibit dual roles in OC progression through senescence–immunometabolic crosstalk. Emerging experimental evidence delineates their functional specificities. HMGB3 acts as a molecular rheostat balancing genomic stability and stemness. In OC models, HMGB3 knockdown triggers PARP1 trapping at DNA damage sites, suppressing its auto-PARylation activity and enhancing replication stress-induced apoptosis [44]. Conversely, HMGB3 overexpression activates MAPK/ERK signaling to promote cancer stemness and chemoresistance [45], suggesting a dosage-dependent role in senescence escape. Mechanistically, HMGB3 maintains ATR/CHK1-mediated DNA damage response [46], potentially explaining its protective association in low-risk tumors. MITF drives OC stemness by hijacking senescence checkpoints. Functional studies demonstrate MITF upregulates CDK2/Cyclin D1 while suppressing E-cadherin, enabling unrestricted proliferation and epithelial–mesenchymal transition [47]. This aligns with our observation of MITF’s positive correlation with high-risk scores, where tumors exhibit stem-like transcriptional programs. CKAP2 promotes cytoskeletal remodeling linked to aggressive phenotypes. OC-specific studies confirm CKAP2 overexpression activates FAK-ERK2 signaling to enhance proliferation/migration [48]. Though direct OC evidence is limited, CKAP2’s mitotic regulatory role (microtubule dynamics/chromosome segregation) [49,50] suggests potential synergy with senescence bypass mechanisms—a hypothesis requiring OC-specific validation. STAT1 exhibits context-dependent immunoregulatory functions. While high STAT1 expression correlates with favorable prognosis in OC [51], its activation upregulates PD-L1 via IFN-γ/STAT1 signaling [52], potentially contributing to the immune-evasive TME observed in C2 subtypes. This dual role underscores the need for spatial characterization of STAT1+ cell populations. ME1 modulates metabolic adaptation in therapy resistance. OC transcriptomics links ME1 downregulation to platinum resistance through altered glycolysis–OXPHOS balance [53]. Its co-expression with antioxidant/STEMness genes in fallopian tube epithelia [54] suggests ME1 may preserve redox homeostasis to counteract senescence-associated oxidative stress. CTSD lysosomal activity facilitates protumorigenic microenvironment remodeling. Though OC-specific data are lacking, breast cancer studies show CTSD promotes mTOR-driven proliferation and M2 macrophage polarization, mechanisms compatible with the pro-metastatic TME features of our high-risk cohort [55,56]. Targeted inhibition studies in OC models are urgently needed.

TMB, defined as the total somatic mutations within a tumor genome [57], serves as a critical determinant of tumorigenesis, progression, and therapy response [58]. Contrary to the conventional paradigm linking high TMB to improved survival via enhanced immunogenicity [59], our senescence-centric risk stratification revealed a counterintuitive TMB-prognosis relationship in OC. The low-risk cohort exhibited significantly higher TMB levels than the high-risk group (*p* < 0.05), with elevated TMB correlating with favorable outcomes. This paradox suggests that low-risk tumors may harbor a unique “immunogenic fingerprint” that augments neoantigen presentation and immune recognition—a phenomenon potentially mediated by GPCR and neuroendocrine pathway activation, which could synergize with TMB-driven immunogenicity to suppress malignancy [60,61]. Conversely, high-risk tumors, characterized by ECM remodeling and immune dysregulation, likely evade TMB-related immune surveillance through stromal barrier formation and checkpoint-mediated T-cell exhaustion [62,63]. These findings redefine TMB’s contextual role in OC, positioning it as a biomarker whose prognostic value is modulated by senescence-related microenvironmental cues. Targeting ECM-immune crosstalk in high-risk tumors or leveraging GPCR/neuroendocrine signaling in TMB-high low-risk cases may unlock novel therapeutic avenues.

In the exploration of the immune landscape, we detected that the high-risk patient cohort manifested a significant elevation in the infiltration levels of a diverse array of immune cell populations, particularly iDCs and cytotoxic cells (*p* < 0.05). Moreover, TME scoring indicated that the high-risk cohort exhibited elevated immune, stromal, and ESTIMATE scores. While high immune infiltration is often considered indicative of anti-tumor activity, it may, in fact, reflect tumor-driven inflammatory responses [64]. The infiltrating immune cells may not effectively target and kill the tumor; rather, they could be co-opted by cancer cells to promote tumor proliferation and metastasis [65,66]. Therefore, it is crucial to precisely analyze the functional state of immune cells, block pro-tumor signals, and reshape the immune microenvironment.

The drug sensitivity analysis underscores the guiding value of risk stratification in medication selection. Docetaxel and Olaparib exhibited higher IC50 values in the high-risk group, suggesting a potential risk of drug resistance, which may be linked to specific gene expression patterns and pathway activation. Risk-based drug selection could help avoid ineffective treatments and reduce patient burden. In the future, drug-gene interaction networks could be developed to predict individual drug responses, facilitating personalized chemotherapy and targeted therapies.

Despite these advances, limitations warrant consideration. While external validation in GSE26712 supports generalizability, prospective multi-omics cohorts are needed to control for batch effects and therapy heterogeneity. The model’s moderate AUC reflects challenges in capturing senescence heterogeneity through bulk RNA-seq—a limitation addressable via spatial transcriptomics or single-cell sequencing. Furthermore, while computational drug sensitivity analysis provides mechanistic hypotheses, ex vivo validation using patient-derived organoids remains essential.

## 5. Conclusions

Our study establishes cellular senescence as a pivotal regulator of OC progression, with CSGs influencing TME remodeling, metabolic adaptation, and therapeutic response. The risk-stratified model and molecular subtyping framework provide actionable insights for personalized therapy: C2/high-risk patients may benefit from combined immune checkpoint blockade and stromal-targeting agents, while low-risk patients could leverage GPCR-targeted immunomodulation. Prospective validation of these findings in clinical trials is warranted to translate senescence biology into precision oncology.

## Data Availability

The TCGA and GTEx integrated data were downloaded from Xena (https://xenabrowser.net/datapages/) (accessed on 22 December 2024). The CSGs were downloaded from the CellAge database (https://genomics.senescence.info/cells/index.html) (accessed on 22 December 2024). Immunostaining images were collected from the Human Protein Atlas (https://proteinatlas.org/) (accessed on 28 December 2024). The GSE26712 dataset was downloaded from GEO (https://ncbi.nlm.nih.gov/geo/) (accessed on 22 December 2024).

## Supplementary Materials

The following supporting information can be downloaded at: https://www.mdpi.com/article/10.3390/biomedicines13040877/s1. Table S1: Cellular senescence-related genes. Table S2: Differentially expressed cellular senescence-related genes. Table S3: TCGA_OV patients’ IDs for molecular subtypes of cellular senescence. Figure S1: Distribution of CSG subtypes (C1/C2) across TCGA molecular classifications. Figure S2: Stratified survival analysis of C1 versus C2 within TCGA subtypes.

## Abbreviations

The following abbreviations are used in this manuscript:

OC	Ovarian cancer
SASP	Senescence-associated secretory phenotype
TME	Tumor microenvironment
CSGs	Cellular senescence-related genes
CNV	Copy number variation
FC	FoldChange
DEGs	Differentially expressed genes
OS	Overall survival
GO	Gene Ontology (GO)
KEGG	Kyoto Encyclopedia of Genes and Genomes
DE-CSGs	Differentially expressed cellular senescence-related genes
PCA	Principal component analysis
GSEA	Gene Set Enrichment Analysis
LASSO	Least Absolute Shrinkage and Selection Operator
K–M	Kaplan–Meier
TMB	Tumor mutation burden
IC50	Half-maximal inhibitory concentration
ECM	Extracellular matrix
DC	Dendritic cell
iDC	Immature dendritic cell
AUC	Area under curve
GPCRs	G-protein-coupled receptors
