# Supplementary material for: Integrative Bioinformatic Analysis of Cellular Senescence Genes in Ovarian Cancer: Molecular Subtyping, Prognostic Risk Stratification, and Chemoresistance Prediction
Source: Biomedicines. 2025 Apr 4;13(4):877. doi: 10.3390/biomedicines13040877 (PMC12025183; doi:10.3390/biomedicines13040877)
Supplement: Supplementary file 1 [file biomedicines-13-00877-s001.zip › Supplementary Materials.zip!/Supplementary Materials.pdf]

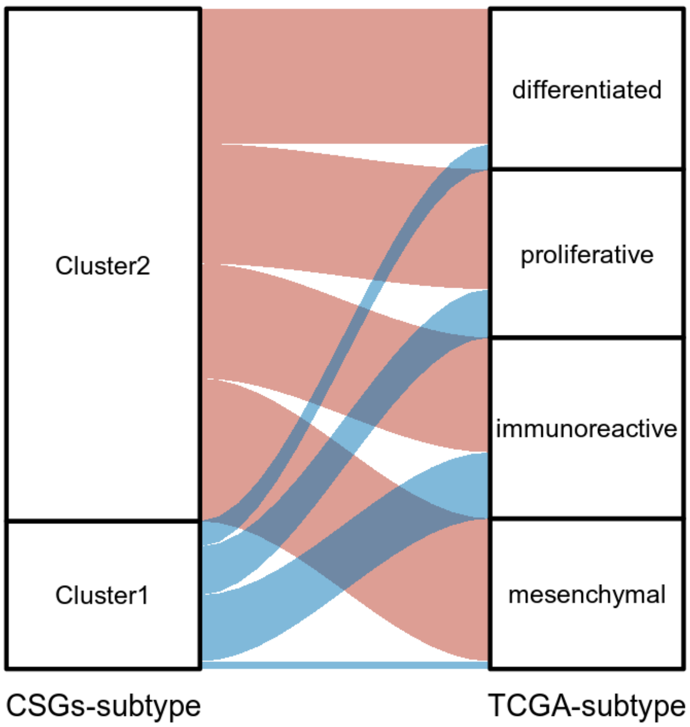

Figure S1. Distribution of CSG subtypes (C1/C2) across TCGA molecular classifications.

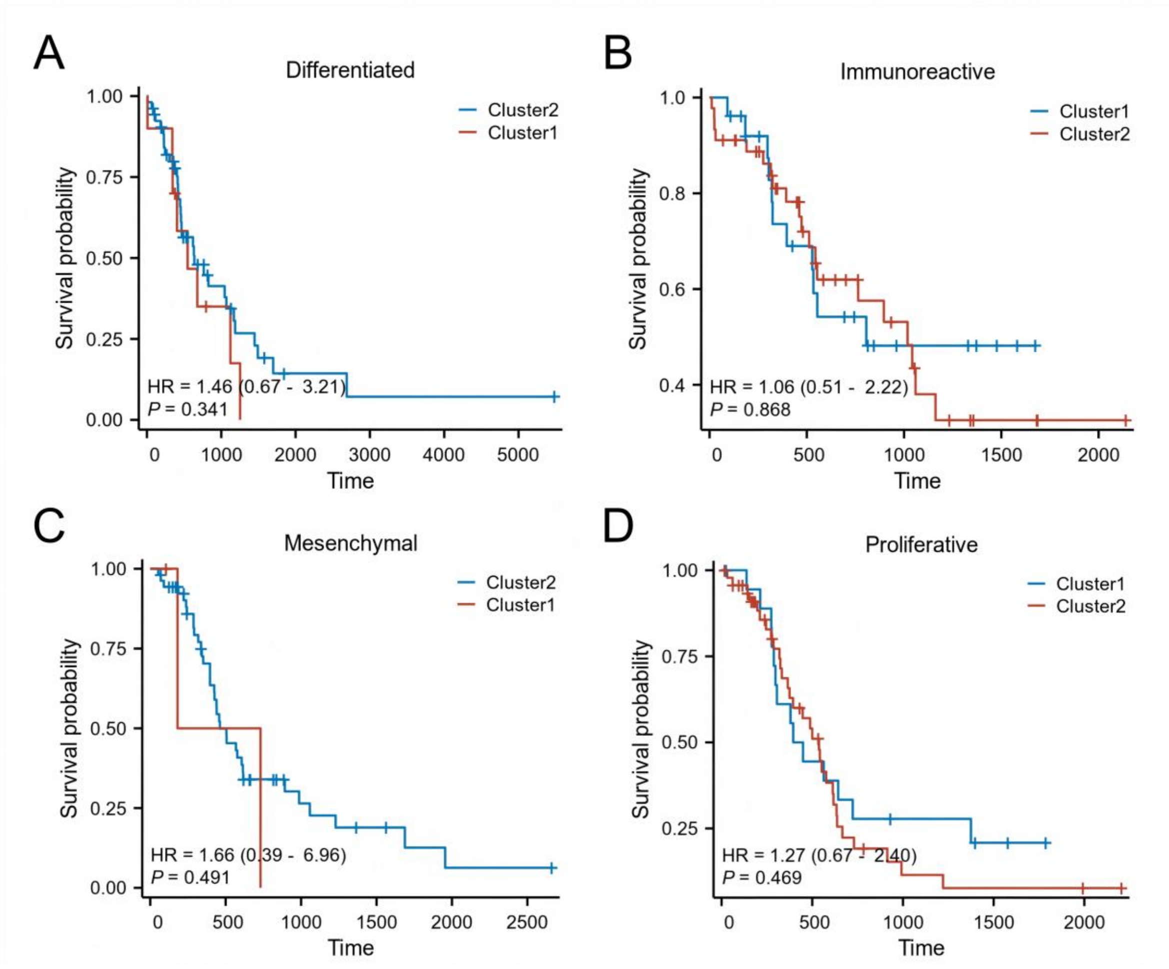

Figure S2. Stratified survival analysis of C1 versus C2 within TCGA subtypes.
